# Embodied artificial evolution: Artificial evolutionary systems in the 21st Century

**DOI:** 10.1007/s12065-012-0071-x

**Published:** 2012-04-20

**Authors:** A. E. Eiben, S. Kernbach, Evert Haasdijk

**Affiliations:** 1 VU University Amsterdam, Amsterdam, The Netherlands; 2 University of Stuttgart, Stuttgart, Germany

**Keywords:** Evolution, Evolutionary computing, Embodiment, Embodied evolution, Self-reproduction

## Abstract

Evolution is one of the major omnipresent powers in the universe that has been studied for about two centuries. Recent scientific and technical developments make it possible to make the transition from passively understanding to actively using evolutionary processes. Today this is possible in Evolutionary Computing, where human experimenters can design and manipulate all components of evolutionary processes in digital spaces. We argue that in the near future it will be possible to implement artificial evolutionary processes outside such imaginary spaces and make them physically embodied. In other words, we envision the “Evolution of Things”, rather than just the evolution of digital objects, leading to a new field of Embodied Artificial Evolution (EAE). The main objective of this paper is to present a unifying vision in order to aid the development of this high potential research area. To this end, we introduce the notion of EAE, discuss a few examples and applications, and elaborate on the expected benefits as well as the grand challenges this developing field will have to address.

## Introduction

This is a position paper about what we call embodied artificial evolution. Perhaps the best way to introduce this vision is to follow a historical perspective concerning the notion of evolution. 1

In the nineteenth century the theory of evolution was put forward to explain the emergence of Life on Earth. Thus, originally, evolution was a passive notion that helped us understand things. In the twentieth century the invention of the computer made it possible to create worlds where we could actively engineer evolutionary processes. The resulting field, called Evolutionary Computing, was groundbreaking in that it converted evolution from a passive explanatory theory to clarify a past process into an active tool to create a new process. Of course, such an evolutionary computing process takes place in an imaginary space, while natural evolution takes place in the biosphere on Earth. And thus, the birth of Evolutionary Computing represents another major transition, that of transporting evolution from biological spaces to digital spaces.

Evolutionary Computing has radically changed the way we think about evolution and it has enabled us to play around with it. We have constructed various forms of evolvable digital objects. We have invented and tested various selection and variation mechanisms, including ones that do not exist in Nature, e.g., crossover mechanisms between more than two parents [30]. And we have designed numerous evolutionary algorithms inspired by natural mechanisms, but not limited by constraints of physical or biological reality. All in all, we have learned a lot about how to set up and to control evolutionary processes and have developed the know-how to use them for solving optimisation, design, and modelling problems [23, 29].

To date, the one space where we can design, implement, and execute all components of an evolutionary process—in a simplified form—is inside computers, in digital space. Therefore, the only type of evolution that we fully master is inherently disembodied. However, in some cases the result of such a digital evolutionary process can be constructed physically. Hence we have two principal kinds of applications. In the first kind, the evolutionary process and the result are both digital. Well known areas in this category are evolutionary optimisation, evolutionary data modeling and evolutionary simulations in artificial life, evolutionary economy, etc. [4, 35, 60]. In the second kind, the evolutionary process is digital, but the result of evolution (e.g., the blueprint of a chair or an antenna) is made physical by an extra construction step afterwards. This is known as evolutionary design with evolutionary art as a special sub-area [7, 8]. Recent advances in rapid prototyping (3D printing), material science, soft robotics, molecular engineering, synthetic biology, combinatorial chemistry, programmable matter, etc. now open the door to creating evolvable objects and to implement evolutionary operators in physical space. This enables artificial evolution of the third kind, where the evolutionary process and the result are both physical. The resulting system means a radically new use of evolution as a tool in a physical medium. From the historical perspective, this will be the twenty-first century variant defined by two essential features: It is fully embodied—similar to biological evolution—and artificially engineered—similar to evolutionary computing. Hence the name Embodied Artificial Evolution (EAE).

In this article we argue that EAE forms a high potential research and application area that offers great opportunities and poses great challenges. However, to realize the vision, very diverse and presently segregated fields need to interact and cross-fertilize each other. This necessitates a unifying view, corresponding terminology, and vision to catalyze developments in this direction. This is exactly the main objective of this paper.

## What is embodied artificial evolution?

The general concept of EAE as assumed here is independent from the specific form of embodiment. One can think of cell-like structures in a liquid solvent, a population of robots exploring another planet, or anything else, as long as the given system satisfies the following properties:

1. It involves physical units instead of just a group of virtual individuals in a computer.
2. It has real ‘birth’ and ‘death’, where reproduction creates new (physical) objects, and survivor selection effectively eliminates them.
3. Evolution is driven by environmental selection or a combination of environmental fitness and a user defined task-based fitness.
4. In contrast to mainstream evolutionary computing, reproduction and survivor selection are not coupled. They are not executed through a centrally orchestrated main loop, but in a distributed manner, controlled by the individuals who ‘decide’ themselves when and with whom to mate.

Observe that in terms of evolutionary computing these properties concern representation, variation, selection, and population management. Furthermore, it can be noted that it is properties 1 through 3 that represent the physical embodiment. The fourth feature smoothly fits this set of properties and it is literally more natural than centrally controlled population management. However, in a strictly formal sense, it is not necessary for being embodied.

To aid further elaboration, we consider a number of concrete examples and tasks and use these to illuminate some important properties of EAE systems.

1. The evolutionary design of a robot controller for a given robot body and some task(s) in a certain environment. Here, the objects to be evolved are digital, but are inherently part of a (mechatronic) physical entity. To solve this design problem one could port all evolutionary operators to the robot and execute on-the-fly evolution of controllers. Birth and death, i.e., reproduction and survivor selection, is restricted to the digital space of all possible controllers, on the robot’s processors. However, fitness evaluation happens in vivo here as the reproductive probabilities of any given controller are determined by the real-world performance of the robot driven by that controller.
2. The evolutionary design of a robot body for some task(s) in a certain environment. 2 Here, the objects to be evolved are physical. Thus, one could solve this problem by truly embodied evolution, with physical birth and death. In such a system all evolutionary operators work in vivo, including reproduction that creates new robots and survivor selection that effectively eliminates them. The main challenge here is obviously formed by the reproduction operators crossover and mutation: how to engineer a system where robots can be born (and die)?
3. The evolutionary design of a bacterium for some medical or chemical task(s) in a certain environment. Here again, the objects to be evolved are physical. However, while (re)production of mechatronic bodies is a huge challenge, bacteria reproduce by themselves. Thus, that part of the evolutionary machinery is for free in this context. The challenge here is to implement fitness evaluation and the selection operators suited to the given application objectives. Furthermore, one could implement special reproduction operators (mutation and/or crossover) that do not exist in nature, but are useful to solve the given problem.

We can note a couple of things about these examples that help understand some essential aspects of EAE. To begin with, observe that Example 1 is different from Examples 2 and 3 in that it is not truly embodied. To be specific, Examples 2 and 3 illustrate applications where the objects to be evolved are physical. In contrast, the objects to be evolved in Example 1 are digital, only embodied in the sense that they are hosted by a physical robot. Ironically, the term embodied evolution has been introduced for systems like the one in Example 1, cf. [98]. If needed, we can make a distinction by calling this type of systems weakly embodied and using the term strongly embodied for the ones in Examples 2 and 3.

Furthermore, let us note that in case of a robotic application it is possible to separate the body, i.e., the physical robot with its wheels, sensors, etc. and the mind, i.e., the controller regulating the behavior of the robot. Consequently, the task of designing them also can be split in two (and combined, if needed). For the task of designing bacteria, this is not possible, because the regulatory and control mechanisms in bio-chemical organisms are not separated so clearly from the bodies to be regulated.

Yet another difference between a robotic application and a bio-chemical one is the fact that a robotic object is more controllable for the experimenter. Robot bodies are built and robot controllers are programmed by the human experimenters. Even if we consider evolutionary development of robot bodies and controllers, the process is driven by human designed operators. These operators are usually simple; complexity emerges by their interactions. This is not the case for bio-chemical organisms, where the operators are those invented by nature. These are often very complex to understand and to manipulate. For instance, replacing one mutation operator by another one can be easy in an evolutionary robotics application, but switching off one molecular interaction and switching on another one in a cell can be (nearly) impossible.

## Motivations, expected benefits

A straightforward motivation to use a technology is that it is $\dots$ useful. Considering breeding livestock or plants as EAE systems (technically: artificial selection and natural reproduction in an embodied setting) we can argue that their usefulness has already been proven. As for the new kind of EAE systems we advocate here, there are multiple reasons to investigate them.

**Fig. 1:**
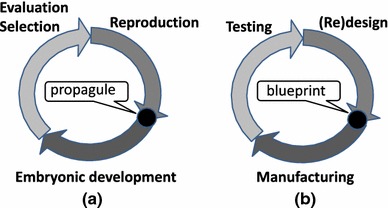
*Two circles* showing the analogies between the biological circle of reproduction (**a**) and the new kind of in vivo evolutionary design (**b**). The effective lifetime is captured by the *light gray* arrow labeled “Evaluation, selection” and “testing”, respectively

Firstly, EAE can lead to solving new design and engineering problems, and solving existing ones in new ways. In fact, EAE technology can be the basis of a paradigm change in how design tasks are solved. Traditionally, the design process of some artifact ends with manufacturing it. Using EAE, design and manufacturing become an intertwined, continuous, on-line activity, propelled by the evolutionary operators (see Fig. 1 and the example application in Sect. 5.3). In the long term, the basic design-and-manufacture loop of the production industry may be transformed from the present off-line type with a critical role for the human designer to a more on-line process. In this process new designs arise though evolutionary variations (are ‘born’), tested immediately in vivo, and reproduce to seed new designs, if successful. While this is clearly not an appropriate workflow for all production industries, there are several potential application areas ranging from fashion items to bio-medical nano-robots.

Secondly, there is much evidence in traditional evolutionary computing that evolution can solve problems not solvable otherwise and that evolution can generate unexpected solutions. (Which, then, can be analysed and reverse-engineered, and thus lead to new insights and better understanding of the problem.) Well-known examples of evolution outperforming human experts or surprising researchers range from Keane and Brown’s experiments in satellite boom design [52] to Koza et al.’s inventory of human competitive genetic programming results [58]. Once we equip certain groups of artifacts with the ability to evolve, we create the possibility that some of the evolved designs may be truly original, stepping out of the box with respect to human thinking.

Thirdly, EAE systems can provide a basis for a new experimentalism in biology, where evolution can be studied in a radically new way in a new medium. To this end it is worth noting that mankind has thousands of years of experience with artificial selection, for instance to breed livestock or plants. As mentioned above, technically speaking this amounts to artificial selection and natural reproduction applied to natural bodies and it has been a valuable tool in using as well as understanding biology and evolution. The new kind of EAE systems we envision extend this in two important ways: artificial evolvable objects (bodies) and artificial reproduction operators (mutation and recombination). The resulting artificial evolutionary systems offer tools to perform real-world evolutionary experiments that are controllable, repeatable, and (relatively) fast, challenging current thinking about the evolutionary process per se. This will enable a deeper understanding of evolution in general, not restricted to or constrained by evolution-as-we-know-it based on our only example, life on Earth. Mastering all components of the system enables us, for instance, to investigate the minimum requirements of evolution, to estimate how (un)likely evolution is, to distinguish different types of evolution, etc. In the long term, this will lead to new scientific insights regarding evolution and the origins of life.

Finally, EAE systems represent a great challenge from the perspective of algorithm design. The twentieth century science/art of designing and analysing evolutionary algorithms needs to be reinvented, once we change the medium from purely digital to embodied, physical. The fundamental problem lies in the inevitable physical restrictions concerning the representation, the algorithmic operators, and the limited options a user has in controlling the algorithm as a whole. Simply put, in evolutionary computing experimenters have great freedom in choosing any data type to represent candidate solutions and defining suitable mutation/crossover operators [23, 29]. However, in an EAE system the bodies to be evolved and the reproduction operators must be physically viable. Further to operator design, we also face the problem of process control. Just to mention one thing, population size management is trivial in a genetic algorithm, but keeping an evolving population of robots or bacteria from extinction as well as from explosion can be a hard nut to crack [99]. Furthermore, EAEs mean a great paradigm shift from evolving digital objects to evolving things in the real world. This implies that the environment where evolution takes place becomes orders of magnitude more complex with inherent randomness (“the noise and the physics are for free”) and a dynamics never encountered in traditional evolutionary computation. In fact, we can say that adopting this new technology, digital algorithm design will become physical process design, where the convenient distinction of algorithm components (representation, variation operators, selection operators, population management) may not be applicable at all. All in all, EAE represents a new angle for Evolutionary Algorithms for three main reasons: (1) the design of the evolvable objects (representation) and the evolutionary operators is constrained by physical restrictions, (2) process control is much harder as we are not the superusers or omnipotent system administrators in real life, (3) the dynamics, noise etc. of the real world is much more complex than in digital spaces.

## Relevant research areas

We distinguish four possible scenarios for realisation of embodied artificial evolution: micro-/nano- mechatronic, top-down bio-synthetic, bottom-up chemo-synthetic and hybrid ones. In this section, we briefly describe the current state-of-the-art research for each of these areas.

### Micro- and nano-mechatronic systems, evolvable hardware

Mechatronic systems are attributed to different areas of robotics [54, 92]. In the context of EAE, the embodiment [77] of robotic systems (using specific properties of materials to achieve a desired functionality, e.g., locomotion for small jumping robots [57] or embodied sensor-actor coupling [53]) has a decisive role. Modern robotics utilises different fields of material science, e.g., [44], which vary from modifications of surface properties up to composite materials with specific mechanical features; miniaturisation of micro-systems [71] and structuring of material by micro-/nano- manipulation [36, 71]. To underline these research areas, we denote this scenario as micro- and nano- mechatronics. The relevance to EAE lies in three approaches: using stand-alone robots for exploring situated evolution, creating a programmable mechatronic matter through guided self-assembling and non-biological self-reproduction.

In the literature various references can be found to work related to EAE in a population of stand-alone robots for exploring evolutionary properties of such systems [41]. Watson et al. in [39, 98] envisioned embodied evolution: a “large number of robots freely interact with each other in a shared environment, attempting to perform some task". In this sense, a population of individuals (in this case, robots) evolves in a completely autonomous manner, i.e., evaluation, reproduction and selection operators are carried out by and between individuals themselves. As in natural evolutionary systems, adaptive mechanisms are asynchronous, decentralised and distributed. Schut et al. [88] present a related concept called situated evolution, where reproduction creates new minds that become active in a pre-existing robot body, replacing an old one. 3 In [95], Usui and Arita address embodied evolution as in Watson et al.: robots evolve based on interactions with the environment and other robots. Nakai and Arita [70] extend this framework by introducing a pre-evaluation mechanism, intended to restrain robot behaviours that are estimated to be have a low fitness contribution. Following then same argumentation, Elfwing et al. in [33] also make use of a subpopulation of virtual agents for each (physical) robot in order to overcome the restriction on population size.

Another state of the art approach applies evolutionary operators not only to the robot controllers but to the robots themselves. In this case the body of the robot has a modular structure and is created through self-assembling process guided by evolution. Multiple research projects, such as HYDRA [50], Molecubes [104], Polypod [102], M-TRAN [51], SuperBot [91], SYMBRION [62] develop heterogeneous reconfigurable platforms. A number of publications are devoted to application of evolutionary approaches [93] or guided self-assembling [55] to create a body of modular robots. Not just the evolution of robot’s body, but also the co-evolution of body and mind is an important aspect of such research [78]. The general technological trend here is to switch from current mini-scale modules to micro- and potentially to nano-scale elements [87]. In the context of body-mind evolution, the concept of evolvable hardware [45] needs to be mentioned. Flexibility and a developmental plasticity of such devices allow deriving an advanced computational functionality in hardware [47], which is used in robotics, image processing and other technological areas. Several open issues in the development of evolvable hardware are in discussion, e.g., [26].

Finally, self-reproduction of micro- and nano-mechatronic systems is of interest for EAE. One of the oldest ideas is Von Neumann’s kinematic self-reproduction [97]. There are multiple attempts to create macroscopic self-reproduction, e.g., by NASA [42] or in the context of modular robotics [104]. They argue that mechanical self-reproduction is possible and not unique to biology. Recent works attribute capabilities of self-reproduction to nano-technological systems [61], to additive plastic moulding [90] (see also RepRap.org), or to advanced 3D prototyping technology [82]. However, none of these technologies is capable of reproducing complex functional elements, see Sect. 6.2.

### Top-down bio-synthetic systems

Biological systems have an advantage over mechatronic devices because biological properties, such as reproduction, can be taken for granted: a biological system is naturally equipped to carry out evolutionary processes. Reproduction, self-preservation, but also selection and adaptation are inherent capabilities of the system. However, an important challenge is how one can manipulate the system to obtain the behaviour one is looking for. Programming cells does not aim to substitute silicon computing, but seeks access to the numerous functionalities and properties on those cells in a predictable, reliable way.

Advances in the area of synthetic biology have allowed some interesting recent results. For instance, in [94], Tamsir et al. show how logic gates can be built in *Escherichia coli* cells and how complex computations can be produced by “rewiring" communication between cells [94]. Works in this area are related e.g., to a development of bacterial systems [64], genome engineering [14], or molecular synthesis of polymers [76]. Intensive research is also devoted to biologically engineering multi-cellular systems [6]; see more about general fields and challenges of synthetic biology in [2].

In biological computing, natural processes can be often described in terms of a networks of simple computational components, or biobricks [3]. The main objective is to use the power of natural processes for the purpose of computation. Because natural processes are intrinsically random, changing functionalities of a cell, as well as adding new desired behaviours is not a trivial exercise. Using an alternative approach, Rigot et al. describe in [80] how to implement complex Boolean logic computations, which reduces wiring constraints. This is obtained through a redundant distribution of the desired output among the engineered cells. Following the idea of biobricks, a number of cells can be combined into more complex circuits.

### Bottom-up chemo-synthetic systems

The bio-synthetic systems utilise existing biological cellular systems with their very complex metabolism. The approach from bottom-up chemistry uses another methodology: creating elementary basic cellular (so-called vesicles) and multi-cellular structures “from scratch". Advantages of this approach are multiple degrees of freedom in designing metabolic networks (in simple cases – autocatalytic reactions) and different internal and external interaction mechanisms [68].

Examples of bottom up chemical systems can be found in artificial chemistries [25], self-replicating systems [49], using bio-chemical mechanisms for, for example, cognition [21]. This approach is also denoted as swarm chemistry [85]. Researchers hope that such systems will answer questions related to developmental models [5], chemical computation [9], self-assembly, self-replication, and simple chemistry-based ecologies [10] or that they will yield technological capabilities for creating large-scale functional patterns [103]. Several approaches consider meso- and nano-objects, such as particles with functionalised surfaces [86], colloidal systems [43], or molecular networks [73]; a system of elementary autonomous agents, which possess rudimentary capabilities of sensing and actuation. Information processing and collective actuation are performed collectively as, for example, stochastic behavioural rules. Several phenomena, such as meso-scale self-assembling or diverse self-organising processes [22], make these type of systems attractive in applications. L. Cronin et al.’s work with polyoxometalate clusters provides an example of chemical synthesis of advanced functional materials on both the molecular level and the nano/microscale [17, 20].

For the design of EAE in molecular, colloidal and particle systems, large-scale interaction patterns for whole systems [59] can be used. Projects such as ECCell [15], BACTOCOM [72], MATCHIT [66] or "Behavior-Based Molecular Robotics" [63] are addressing the questions of programmable chemo-ICT interfaces. Essential attention is paid to a self-replication of chemo-synthetic systems [37, 38]. Research in collective nanorobotics is also focused on the technological capabilities of creating such large-scale patterns in molecular systems, e.g., [103].

### Hybrid mechatronic and biochemical systems

Hybrid mechatronic and biochemical systems combine advantages of both types of technologies and are of essential interest for EAE. There are several reasons for this: sufficient computational properties, high developmental plasticity, utilization of natural self-reproduction processes. Examples of hybrid systems are bacterial cellular sensors [101], development of bio-hybrid materials [84], molecular synthesis of biofuels [1]. Another example of hybrid technologies are attempts to interact with biological populations by means of technological artifacts: managing the grazing of cattle over large areas [18, 89], controlling mixed societies of robot and insects [12], or a social communication between robots and chickens [46]. A similar approach is related to the integration of different robot technologies into human societies, for example the management of urban hygiene based on a network of autonomous and cooperating robots [67].

One of the interesting approaches in the area of hybrid technologies is a combination of cultured (living) neurons and robots [74] to investigate the dynamical and adaptive properties of neural systems [79]. This work is also related to understanding of how information is encoded [19] and processed within a living neural network [24]. The hybrid technology can be used for neuro-robotic interfaces, different applications of in vitro neural networks [69] or for bidirectional interaction between the brain and the external environment in the EAE system. Several research projects, e.g., NeuroBit, already addressed the problem of controlling autonomous robots by living neurons [65].

## Applications

The proof of the pudding is in the eating: new technology is largely justified by useful applications. In the present embryonic stage of the EAE field, it is impossible to predict what the best applications will be. To this end, we see an analogy with the first decade(s) of the computer industry in the 1950s. This was when when an IBM executive foresaw a world market for perhaps 5 computers all together. Half a century later, there are more computing devices than human beings and countless applications that one could not imagine in the early years of the technology.

As for EAE, we are at the down of the technology, and we dare not predict specific applications. Hence, in the rest of this section we just briefly discuss some potential application areas. In general, EAE systems are suitable for the design and production of artifacts under complex circumstances, for instance in case of (1) changing environments, (2) unforeseen environments, (3) ill-defined (implicit) objectives, or (4) multiple objectives with complex interactions (possibly conflicting). Furthermore, we can distinguish between artifacts that are passive, e.g., jewelry, and those that are active, e.g., micro-robots. These two types differ substantially in that active objects need an inner controller to govern their behavior, while passive ones do not. With a biological analogy we may say that passive objects need a only a body, while active ones need a body and a mind.

### Evolving robots

One could imagine whole ecosystems of robots on different scales of size. On a very small scale we could have medical nano-robots to be deployed in a human body. For example, they could be used as “personal virus scanners”, evolving to the metabolism of the host and adapting to fight any new threat be it a germ of cancer. On a larger scale, evolving robot populations for planetary exploration could be interesting. These could be sent to other (unknown) planets with just a rough initial design but with the ability to evolve to the given circumstances. This will enable them to perform exploration and maybe even build a base station from the locally available resources. Still on the large scale, we can conceive evolving robot companions in domestic and industrial environments. Regarding their bodies, these could range from cat and dog size up to human comparable sizes. As for their mental features, they should be human-friendly and intelligent. From a functional point of view they should perform specific tasks and in a domestic setting they could provide more generic ‘emotional’ services (keeping company, being good listeners, acting as partners in simple conversations) [100].

### Functional organisms

April 2010 saw the largest oil spill in US history: the equivalent of around 4 million barrels of oil flowed into the Gulf of Mexico, with numerous ecological implications. Analysis on the site, a couple of weeks after the disaster, showed that many groups of bacteria were helping to clean up the waters. These bacteria were able to break down the chemicals found in crude oil and, in fact, responded quite effectively to the incident. In general, there are many possible applications of bacteria, or some other type of organism, that are synthetically designed for a specific functionality. Such artificially developed organisms can be used, for instance, to provide environmental services, create building material or biofuel, to store data, or to stop desertification. An evolutionary approach is literally natural in this application area. At this moment, this line of research—positioned in synthetic biology—is perhaps the closest to a breakthrough, cf. Sect. 4.2.

### Evolutionary personal fabrication

Imagine a world in which anything can be produced with just a few clicks. Customised products are at the reach of your hand, ranging from a child’s toy to a meal. Vilbrandt et al. introduce in [96] the idea of *universal desktop fabrication* (UDF) that can produce essentially any complete, finished, and functional object. Fab@Home (http://www.fabathome.org) is a desktop rapid prototyper (3D-printer) and a first step towards UDF. Such personal fabricators can build a great variety of objects from different materials and thus enable a large group of people to produce stuff to fit their needs locally. The range of applications is not restricted to solid objects, such as personalised fashion items (jewels, sunglasses, smartphone cases), but may also include consumables, like food: “You can imagine a 3-D printer making homemade apple pie without the need for farming the apples, fertilising, transporting, refrigerating, packaging, fabricating, cooking, serving and the need for all of the materials in these processes like cars, trucks, pans, coolers, etc,". 4 Embodied evolutionary technology is expected to play an important role in the development of such fabricators, cf. [81, 82] : “Ultimately, the evolution of form and formation become fully intertwined when the language of assembly itself becomes subject to evolution […]. Through this co-evolution of form and formation, Evolutionary Fabrication discovers both how to build objects and what to build them out of.”

In general, evolutionary technology can be used on local and global level. Locally, a limited set of users (one person, a family, or a small firm) would represent the fitness function governing evolution. The system could adapt to their preferences advancing customisation. On a global scale, such personal fabricators could be networked to yield an evolutionary system involving billions of users, evolutionary app stores, and almost incomprehensible dynamics.

## Grand challenges

At this moment it is impossible to foresee how this field will develop. However, we are able to identify some of the grand challenges that certainly will have to be addressed.

### Body types

The essence of embodied evolution is the body. To this end, we can distinguish hardware in the broad sense (mechatronic-robotic systems, new materials, etc) and wetware (bio-chemical systems) that may also be hybridised. Regarding wetware, there are two options again: bottom-up, relying on chemistry, or top-down, based on biology. Recent developments in microfluidics, functional fluids, or programmable matter also seem very promising. The first grand challenge is thus to find body types suited for (self-)reproduction. In essence, this means that we need to inject dead matter with a human requested functionality. This question is also known in other formulations, e.g., “programmability of synthetic systems", or “open-ended embodied evolution”, and is one of the key points in understanding principles of synthetic life. It is also addressed by the European bio-ICT initiative and several research projects, e.g., PACE [75] and e-FLUX [27], to name but a few.

Summarizing, one of the principal challenges of EAE is to find physical constructs that are suited to be the evolvable objects forming the population. Technically this requires that they can be produced and reproduced. This is akin to one of the main problems in Evolutionary Computing: how to find a suitable representation, that is, a data structure that can be used for the individuals representing candidate solutions [83].

### How to start: reproduction of functional elements

The implementation of birth (reproduction operators) for human engineered physical artifacts is a critical prerequisite for EAE. These operators must also realise some form of inheritance. The approaches based on mechatronics, chemistry, or biology differ greatly in this respect. (Self-)reproducing mechatronic and chemical units are far from being trivial, whereas reproduction comes for free in biological systems.

As mentioned in Sect. 4.1, in current micro- and nano-mechatronic systems there are two concepts that are crucial for EAE: self-assembling and self-replication. Self-assembling is a process which creates complex systems from basic elements, whereas self-replication means a reproduction of these basic elements. Robots are able to make functional copies of artificial organisms that consist of basic building blocks if they have access to a reservoir of these basic modules. Things are different, however, when it comes to the self-replication of basic modules that contain functional elements such as motors, gears or silicon-based microelectronics: due to their high technological complexity, self-replication of these functional elements remains, to date, an unsolved issue.

### How to stop: kill switch

A serious concern regarding EAE is the possibility of runaway evolution. By this term we do not mean the Fisherian notion of sexual selection reinforcing useless traits [40]. Runaway evolution as we use it here stands for the process of uncontrolled population growth. Such a growth might also be accompanied by the emergence of new, unwanted features in the population. Obviously, it would be highly irresponsible to expose ourselves to such a risk. To reduce this risk, all such experiments could be carried out in highly secured isolated environments, not unlike current research into certain germs, bacteria, viruses, etc. involving bio-hazard. However, this might disable the whole application in cases where the evolving population is inherently free, acting ‘out in the wild’ (robot companions, waste-eating organisms, medical nano-robots in the human body, etc.). In such cases a ‘kill switch’ is required to guarantee that human supervisors are able to shut down the system, if and when they deem necessary.

As of today, the kill switch problem has been already recognized within synthetic biology. There are various approaches to obtain a solution, such as for instance suicide genes, programmed cell death (PCD) and apoptosis [11, 13, 34, 56], just to name a few. A particular challenge stemming from the inherent use of evolution is possibility that an evolutionary systems will find solutions that are well ‘outside the box’ for the human designers of these systems (cf. the originality argument in Sect. 3). It is therefore essential that great care be taken when designing kill switches to ensure that evolution will not be able to circumvent them. In common parlance, we need to prevent the ‘Jurassic Park problem’.

### Evolvability and rate of evolution

It is well-known in biology as well as in evolutionary computing that evolution is a relatively slow form of adaptation. To put it simply, it can take many generations to achieve a decent level of development. Obviously, ‘slow’, ‘many’, and ‘decent’ depend on the application context. For instance, medical nano-robots put to work in a human body should adapt within a few hours to their environment (the patient’s body). In case of sending evolving robot explorers with a rough initial design to Mars, one can wait months for appropriate designs to emerge. In general, we can say that useful EAE systems must exhibit a high degree of evolvability and a high rate of evolution [48]. In practice, they must make good progress in real time: have short reproduction cycles and/or large improvements per generation. The main factors here are the application dependent time requirements and quality criteria that define how progress is measured, and the speed of progress determined by the evolutionary operators.

Building fast evolutionary systems is a nontrivial challenge on its own. Failing to meet this challenge would imply that the real time performance of EAE systems is too low. Ultimately, this could even disqualify the whole approach—at least, for certain applications. In general, the speed of evolution should be used as one of the essential assessment criteria for judging the feasibility of potential applications.

### Process control and methodology

A radical change caused by EAE technology is that design and manufacturing become an intertwined, continuous activity. This poses an unprecedented challenge for maintaining human control during the process. In Evolutionary Computing, on-line control of an evolutionary algorithm is exercised through changing its parameter values on-the-fly [32]. Such control is directed to improving the working of the given algorithm, e.g., increasing its speed or recovering from local optima. In the EAE systems we envision, there is additional challenge: we need to combine open-ended and directed evolution on-the-fly. This means that human users should be able to perform on-line monitoring and steering in line with the given user preferences. This could be perhaps realised by directed selection (akin to breeding) and/or directed reproduction (as in genetic manipulation). On a conceptual level, this requires a new kind of methodology that must contain the traditional elements, such as specification, validation, and tuning [28]. Meanwhile, we have to address the novel aspects, such as the combination of free evolution and specific design objectives. Part of this challenge is the ‘freeze switch’, that is, the ability to recognize if/when the evolving objects have obtained the required properties and stop further evolution without killing the system.

### Body-mind coevolution and lifetime learning

As explained in the introduction of Sect. 5, in general we can distinguish passive and active artifacts. Obviously, an active artifact needs an entity governing its activities. In some life forms, e.g., bacteria, the control and regulatory mechanisms form a unity with the body. In higher life forms, such control mechanisms are augmented with a designated control entity, the mind (the ‘software’), carried by a separate part of the body, the brain (the ‘hardware’). 5 Similarly, in EAE systems active artifacts can have a dual structure with a body and a mind (controller) that must fit the given body. This implies that bodies and minds have to coevolve, they will be subject to reproduction and inheritance. Obviously, we do not know how the reproduction and inheritance mechanisms for bodies will be related to those concerning the minds in any specific EAE system. However, in general it cannot be assumed that the inherited mind will perfectly match the inherited body. Therefore, the system must include the possibility that a newborn object undergoes a lifetime learning process—not unlike baby animals have to learn walking, seeing, etc. soon after birth. Depending on the given EAE system at hand, it may be possible to make individually learned skills inheritable, i.e., to make the system Lamarckian. The ‘Artificial’ in EAE offers a possibly large degree of technical freedom, and experimenters of such systems could make their systems Lamarckian, even though biological evolution is not.

## Final remarks

In this paper we have presented the concept of Embodied Artificial Evolution or the Evolution of Things. The systems we envision are embodied because evolutionary operators (reproduction, selection, fitness evaluation) are implemented in/by the physical objects that undergo evolution. Furthermore, they are artificial because (i) the evolvable objects and the population as a whole are being fabricated and/or programmed to fulfill a certain human purpose, to execute a certain task, 6 and (ii) the evolutionary operators (reproduction and selection) and their particular combination into one working system are human engineered.

We believe that EAE offers a high potential research and application area with exciting scientific and technological challenges. This field is in an embryonic stage, where relevant developments take place within different scientific communities and technological areas that do not naturally interact with each other. At the moment we see three main streams of research towards building EAE systems: top-down, biology-based, bottom-up working from chemistry and ‘head-on’ engineering based on robotics and material science. Furthermore, Evolutionary Computing can play an important role as the field that collected a large body of knowledge about designing, implementing, and executing *all* components of an evolutionary process. We hope that by introducing a unifying vision we can bring all stakeholders together raising awareness of the shared research issues and possible solutions.

Last, but not least, let us mention a particular issue all approaches must address: the related ethical questions. In this respect, several problems have already been noticed in Life Sciences [16]. However, EAE systems based on non living mediums could lead to very similar challenges, be it in different forms. For instance, bio-hazard can turn into robo-hazard. The ethical questions therefore form a clearly horizontal issue, cross-cutting over different disciplines and technical approaches to EAE. One of the main goals of this paper is to create an overarching vision, which in turn could contribute to help research communities and institutions develop a solid system of checks and balances thus making such research a safe enterprise.

## Footnotes

1 For a gentle introduction we also recommend the TEDx talk [31], to be viewed at http://www.youtube.com/watch?v=WJX_wAKhg8A.

2 For the sake of simplicity, let us disregard the design of the corresponding robot controller.

3 Although it may be an oversimplification to view a human body (including the brain) as hardware and the mind as software, we find this distinction helpful when considering the parallel development of bodies and their controllers.

4 Homaro Cantu states in the BBC News article “The printed future of Christmas dinner": http://www.bbc.co.uk/news/technology-12069495.

5 This dualist view is in fact an oversimplification that could be debated. Nevertheless, we find it a helpful distinction that reflects the current robotics and computer technology.

6 This does not exclude open-ended evolution to take place in parallel.
